# Cross-ethnic analysis of common gene variants in hemostasis show lopsided representation of global populations in genetic databases

**DOI:** 10.1186/s12920-022-01220-0

**Published:** 2022-03-25

**Authors:** Abdimajid Osman, Jon Jonasson

**Affiliations:** 1 Department of Clinical Chemistry, University Hospital in Linköping, Ing. 64, Plan 11, 581 85 Linköping, Sweden; 2 Department of Biomedical and Clinical Sciences, Linköping University, Linköping, Sweden; 3 Department of Clinical Genetics, University Hospital in Linköping, Linköping, Sweden

**Keywords:** Hemostasis, Thrombosis, Single nucleotide polymorphism, Population genetics, Gene frequency

## Abstract

A majority of studies reporting human genetic variants were performed in populations of European ancestry whereas other global populations, and particularly many ethnolinguistic groups in other continents, are heavily underrepresented in these studies. To investigate the extent of this disproportionate representation of global populations concerning variants of significance to thrombosis and hemostasis, 845 single nucleotide polymorphisms (SNPs) in and around 34 genes associated with thrombosis and hemostasis and included in the commercial Axiom Precision Medicine Research Array (PMRA) were evaluated, using gene frequencies in 3 African (Somali and Luhya in East Africa, and Yoruba in West Africa) and 14 non-African (admixed American, East Asian, European, South Asian, and sub-groups) populations. Among the populations studied, Europeans were observed to be the best represented population by the hemostatic SNPs included in the PMRA. The European population also presented the largest number of common pharmacogenetic and pathogenic hemostatic variants reported in the ClinVar database. The number of such variants decreased the farther the genetic distance a population was from Europeans, with Yoruba and East Asians presenting the least number of clinically significant hemostatic SNPs in ClinVar while also being the two genetically most distinct populations from Europeans among the populations compared. Current study shows the lopsided representation of global populations as regards to hemostatic genetic variants listed in different commercial SNP arrays, such as the PMRA, and reported in genetic databases while also underlining the importance of inclusion of non-European ethnolinguistic populations in genomics studies designed to discover variants of significance to bleeding and thrombotic disorders.

**Supplementary Information:**

The online version contains supplementary material available at 10.1186/s12920-022-01220-0.

## Background

Hemostasis is a complex and vital physiological process that involves activation of clotting factors as well as blood platelets, clot retraction, fibrinolysis and vascular repair [1]. Hundreds of genes are involved in these events, many of which were evolved in early evolution of the animal kingdom and are found in archaic species predating non-bird dinosaurs [2]. Sequence alterations of these genes can pose risk to either bleeding or thrombotic disorders in humans.

A large number of common and rare genetic variants in hemostasis were identified in the past thanks to modern high-throughput genotyping and sequencing approaches that deciphered the human genome. Some of these variants are associated with altered phenotype and, hence, are accessible from public disease database repositories such as ClinVar [3] and Online Mendelian Inheritance in Man (OMIM) [4]. However, the majority of genetic studies reporting such variants were performed in populations of European descent [5] and therefore lack the diversity required to represent global human populations. It has been speculated that researchers in rich western countries often favor the use of existing and homogenous cohorts to avoid too many confounding variables associated with the study population, resulting in lack of representation from other populations [6]. One study investigating the ethnic diversity of clinically relevant DNA variants reported on ClinVar recently found that the majority of information found in the database is from European-ancestry individuals [7]. The extent of under-representation of non-European populations pertaining to the hemostatic gene variants included in different commercial SNP arrays, or stored in ClinVar is unclear. In addition, large-scale genome sequencing ventures such as the 1000 genomes project [8] and the Genome Aggregation Database [9] lack data for many ethnolinguistic populations in the world, particularly in Africa where most of the human genetic diversity is found. For instance, only 7 out of 26 populations included in the 1000 genomes project represent Africans, and all but 1 of these 7 African populations have ancestry in West Africa [10]. The purpose of this study was to conduct a cross-ethnic examination of common hemostatic gene variants to estimate the degree of mentioned asymmetric representation of global populations.

## Methods

### Populations and datasets

Fourteen populations were included in the analyses: African (Somali in Northeastern Somalia [SOM], Luhya in Webuye, Kenya [LWK], and Yoruba in Ibadan, Nigeria [YRI]), African Americans in Southwest USA (ASW), ad-mixed American (Mexican Ancestry in Los Angeles, CA, USA [MXL] and their super-population admixed Americans [AMR]), East Asian (Han Chinese in Beijing, China [CHB], Japanese in Tokyo, Japan [JPT], and their super-population EAS), European (Northern Europeans from Utah [CEU], Toscani in Italy [TSI], and their super-population EUR), South Asian (Gujarati Indians in Houston, Texas, USA [GIH] and their super-population SAS). Except for Somalis (n = 95), genotype data were accessible from the 1000 Genomes Project (n = 4973) [8]. For the SOM population, data were available from our previous study [11] as mentioned above. In the major part of this study, since African populations host the largest human genetic variation, two geographically and ethnolinguistically distant African populations (YRI and SOM) were included in the study whereas all other non-African populations represented continental or sub-continental populations (AMR, EAS, EUR, and SAS) respectively.

### Variant analysis, inclusion criteria and annotations

The analysis of the Affymetrix Axiom Array was performed according to the protocol described in the AxiomTM 2.0 Assay Manual Workflow User Guide by Thermo Fisher Scientific Inc. (Waltham, MA, USA), as we previously described [11]. Genotype results were recorded in plink format. Only variants included in the Axiom Precision Medicine Research Array (PMRA; Thermo Fisher Scientific Inc.) were used for the analyses. Marker inclusion criteria were: genes involved in coagulation, anticoagulation, fibrinolytic system and their regulators, at least 1% of alternative allele frequencies (AAF) in at least 1 population, and within 1 mega base pairs (Mbps) of a hemostatic gene or a drug response gene associated with hemostasis. Genes involved in platelet function or platelet count were not included. SNPs that fulfilled these selection criteria were further evaluated on the ClinVar database (https://www.ncbi.nlm.nih.gov/clinvar/) [3]. Variants were grouped into five categories according to the ClinVar annotations; benign, likely benign, drug response, variant of uncertain significance (VUS), and pathogenic. Variants not recorded on ClinVar were classified as unknown.

### Haplotype phasing

Haplotype phasing was estimated with the Shapeit v2 method [12]. Duplicate SNP positions were excluded. The resulting files were filtered with respect to the 34 coagulation panel genes included in the study. Populations data for comparison were downloaded in hg19 format from the IGSR ftp site (http://ftp.1000genomes.ebi.ac.uk/vol1/ftp/). In the final analysis, we only included the SNPs that were represented in both the Affymetrix genotype array and the phase-determined population data obtained from the 1000 genomes project phase 3.

### Bioinformatics and statistics

Principal component analysis (PCA) was performed with the R software version 3.6.1 [13] using factoMineR and factoextra libraries [14]. Statistics were carried out with SPSS software version 27 (IBM, Armonk, NY, USA) as well as with the R program. Variant heatmap was generated with R software using heatmaply library [15]. Nonparametric tests were carried out to investigate statistically significant differences between populations, using the SPSS package.

## Results

### PCA

**Fig. 1:**
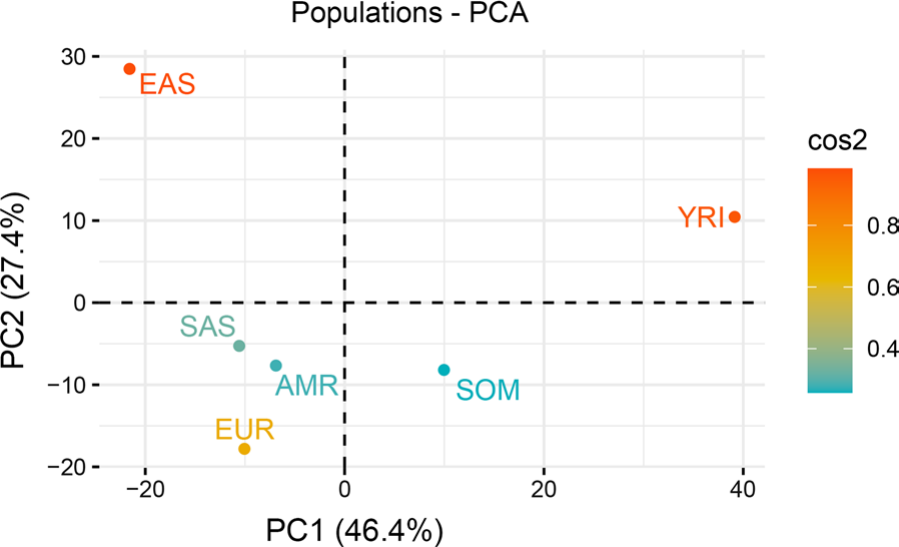
PCA for 845 hemostatic SNPs in 6 global populations. Cos2 values show the quality of representation for the variables on the PCA, the higher Cos2 the better representation

**Fig. 2:**
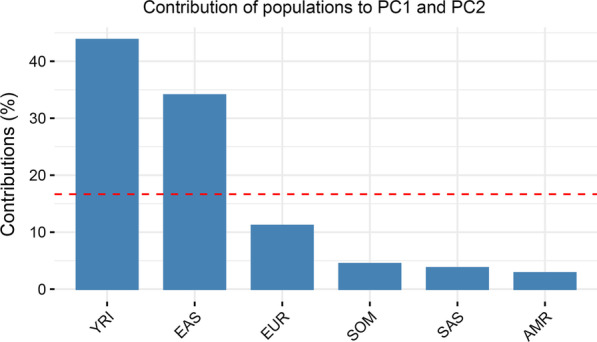
Contributions by each population to the first two dimensions of PCA (PC1 and PC2). The red dashed horizontal line represents the expected mean contribution

Under the predefined inclusion criteria, 845 hemostatic SNPs in 34 genes were found to be eligible for downstream analyses (Additional file 1: Table S1). PCA was performed to infer differentiation of these variants in the populations studied (Fig. 1). The estimated genetic distance to Europeans increased in the order: YRI > EAS > SOM > SAS > AMR > EUR. The largest input to the first two principal components (PC1 and PC2) was made by the EAS and YRI populations, which accounted for nearly three-quarters of the total contributions (Fig. 2). To verify the genetic distance provided by the 845 hemostatic SNPs for populations shown in Fig. 1, we generated another PCA with 849,267 SNPs included in the PMRA. The new PCA confirmed the genetic distance provided by the hemostatic SNPs (Additional file 2: Fig. S1).

### Population allele frequencies and correlations

To investigate whether there were differences in gene frequencies between the populations, allele frequencies for the 845 SNPs were statistically examined over 6 populations (AMR, EAS, EUR, SAS, SOM, YRI) using Kruskal–Wallis test [16]. The Kruskal–Wallis test is a nonparametric test that examines whether groups of populations (*k*) are identical or whether at least one of these populations deviates from other populations. Post-hoc analyses with Bonferroni corrections were then performed to investigate pairwise relationships in gene frequency among the 6 populations. This generated 6 pairs of populations that displayed statistically significant differences in allele frequencies: EAS‒EUR (*P* < 0.05), YRI‒EUR (*P* < 0.05), SOM‒YRI (*P* < 0.05), EAS‒AMR (*P* < 0.001), EAS‒YRI (*P* < 0.001), and SAS‒YRI (*P* < 0.001). No other pairs showed statistically significant differences in gene frequencies. The EAS and YRI populations showed the lowest correlation in gene frequencies between any pair of population compared, with Pearson's correlation coefficient (*r*) corresponding to 0.72 (Additional file 3: Table S3, Additional file 4: Fig. S2). The highest correlation was obtained between EUR and AMR (*r* = 0.96), indicating a significant European admixture in the latter population.

To investigate dispersion of the 845 SNPs over populations, Pearson correlations between allele frequencies of each population and the expected mean allele frequencies for the 6 populations was examined (graphs not shown). The test showed that allele frequencies in Europeans and in populations with genetic proximity to Europeans had the best correlations with the expected mean allele frequencies. The Pearson *r* values then decreased as the genetic distance from Europeans increased in the following order: AMR (*r* = 0.98) > EUR and SAS (*r* = 0.97) > SOM (*r* = 0.96) > EAS (*r* = 0.92) > YRI (*r* = 0.89).

### DNA variants

**Fig. 3:**
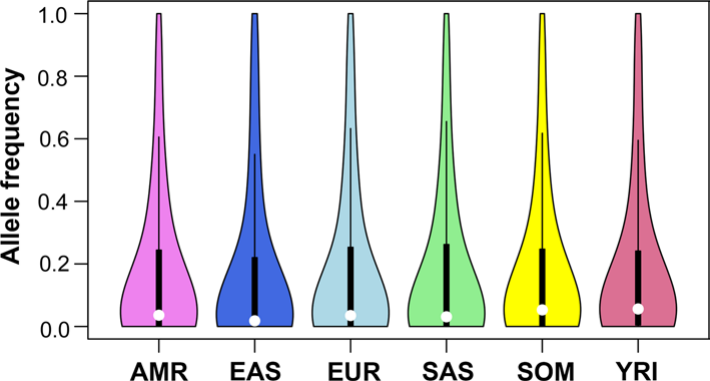
Violin plots showing the distribution of allele frequencies for 845 hemostatic SNPs in different populations. The small white circles indicate the median value: 0.036 (AMR), 0.018 (EAS), 0.035 (EUR), 0.031 (SAS), 0.053 (SOM), and 0.056 (YRI). The black rectangle in the middle represents the interquartile range. Allele frequency refer to the frequency of the alternative allele

In Fig. 3, violin plots are shown that schematically show distribution of allele frequencies for the 845 common hemostatic SNPs. In general, the two African populations (SOM and YRI) had higher median allele frequencies than other populations. The EAS population showed the lowest median allele frequencies among the 6 populations investigated.

**Fig. 4:**
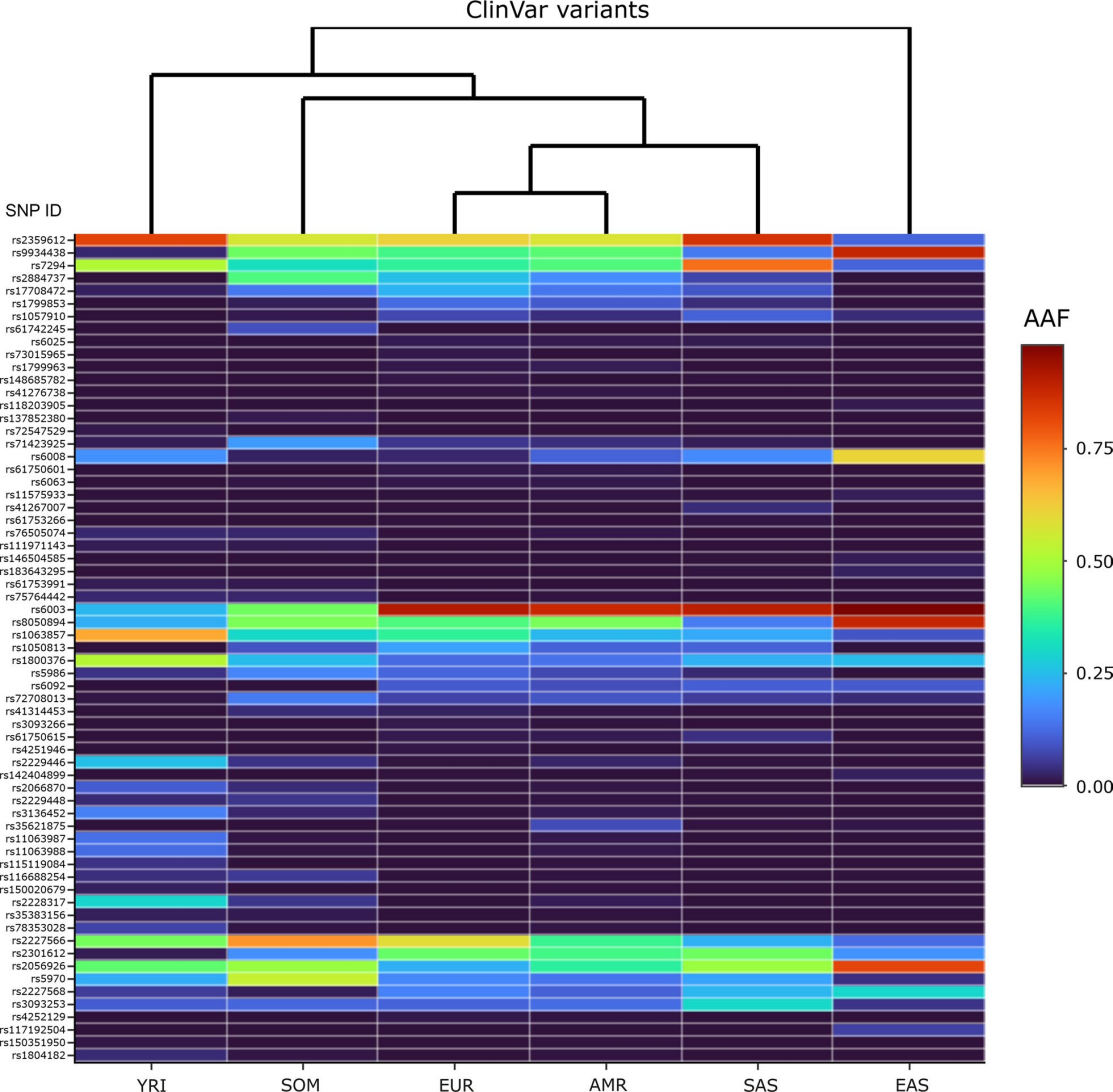
A heatmap showing 66 hemostatic SNPs found in the ClinVar database with at least 1% alternative allele frequency in 6 world populations. The legend on the right side shows corresponding colors for alternative allele frequencies (AAF). The SNPs are arranged from top to bottom as follows according to their ClinVar annotations: drug-response (n = 8), pathogenic (n = 9), variant of uncertain significance (n = 13), likely-benign (n = 26), and benign (n = 10)

Of the 845 hemostatic SNPs included in the analyses, 779 were not recorded in ClinVar and were designated as unknowns. The remaining 66 SNPs were found on ClinVar, of which 10 were annotated as benign variants, 8 as drug response related, 26 as likely benign, 9 as pathogenic, and 13 as variants of uncertain significance (VUS). In Fig. 4, these 66 ClinVar SNPs are shown in a heatmap. It appears in the heatmap that fewer drug response alleles reach frequencies greater than 0.10 in East Asian and West African (YRI) populations (upper segments of the heatmap). Other SNPs, such as the Ala15Thr variant (rs6092) in *SERPINE1* whose pathogenicity status is disputed, have alleles that are prevalent in non-African populations but not in the 2 African populations investigated (see Additional file 1: Table S1).

### Multi-SNP haplotype phasing

To investigate whether multi-SNP haplotype phasing provided better information than individual SNPs, we employed the Shapeit v2 method and constructed haplotypes for the 34 genes included in the analyses. We only included SNPs that were represented in both the Affymetrix genotype array and the phase-determined population data obtained from the 1000 Genomes Project phase 3. Resulting haplotypes are shown in a multi tab Excel file in Additional file 5: Table S2. All reference alleles shown in the table are ancestral alleles. In Table 1 a multi-SNP haplotype phasing for one of those 34 genes – vitamin K epoxide reductase complex subunit 1 (*VKORC1*) – is shown with regard to 8 different SNPs in 10 different populations: SOM, ASW, CEU, CHB, GIH, JPT, LWK, MXL, TSI, YRI. The *VKORC1* gene is shown as an illustrative example partly because of its pharmacogenetic importance (warfarin resistance/sensitivity) and partly because of the high proportion of selected SNPs that given the allele frequencies could be expected to be informative as regards ethnicity. Surprisingly, a mere 8 different multi-SNP haplotypes of *VKORC1* represent the whole spectrum. Those with a frequency less than one percent were excluded (indicated by an empty space in Table 1). The 8 percent ‘C G C C G G A A’ haplotype (haplotype row 6 in Table 1) was unique to the SOM population. To summarize Table 1, it is evident that the multi-SNP patterns of SNPs with high but not complete allelic association (i.e. strong LD plus evidence for historical recombination) in the *VKORC1* gene across ethnic populations are more informative as regards ethnicity compared to looking at SNPs individually.

**Table 1:** *VKORC1* haplotypes in different world populations

Gene	VKORC1								Haplotype frequencies									
Cromosome	16	16	16	16	16	16	16	16	This study	1000 genomes phase III								
Position hg19	31,102,321	31,103,796	31,104,509	31,104,720	31,104,878	31,105,353	31,105,554	31,105,945										
Ref. allele	C	A	C	C	G	G	A	C										
Alt. Allelle	T	G	G	T	A	A	C	A										
rs-number	rs7294	rs2359612	rs8050894	rs72547529	rs9934438	rs17708472	rs2884737	rs61742245	SOM	ASW	CEU	CHB	GIH	JPT	LWK	MXL	TSI	YRI
Haplotypes	C	A	C	C	G	G	A	C		7%					12%			14%
	C	A	C	T	G	G	A	C							3%			
	C	A	G	C	A	G	A	C	3%	8%	17%	95%	11%	90%	3%	33%	15%	3%
	C	A	G	C	A	G	C	C	41%	7%	28%		7%			14%	33%	
	C	G	C	C	G	A	A	C	14%	7%	26%		16%		6%	15%	16%	2%
	C	G	C	C	G	G	A	A	8%									
	C	G	C	C	G	G	A	C	1%	8%					19%			10%
	C	G	G	C	G	G	A	C		15%					13%	3%	2%	19%
	T	G	C	C	G	G	A	C	31%	48%	32%	4%	67%	10%	43%	34%	33%	51%

The following populations were included: Somali in Northeastern Somalia (SOM), African Ancestry in Southwest USA (ASW), Northern Europeans from Utah (CEU), Han Chinese in Beijing, China (CHB), Gujarati Indians in Houston, Texas, USA (GIH), Japanese in Tokyo, Japan (JPT), Luhya in Webuye, Kenya (LWK), Mexican Ancestry in Los Angeles, CA, USA (MXL), Toscani in Italy (TSI), and Yoruba in Ibadan, Nigeria (YRI)

**Fig. 5:**
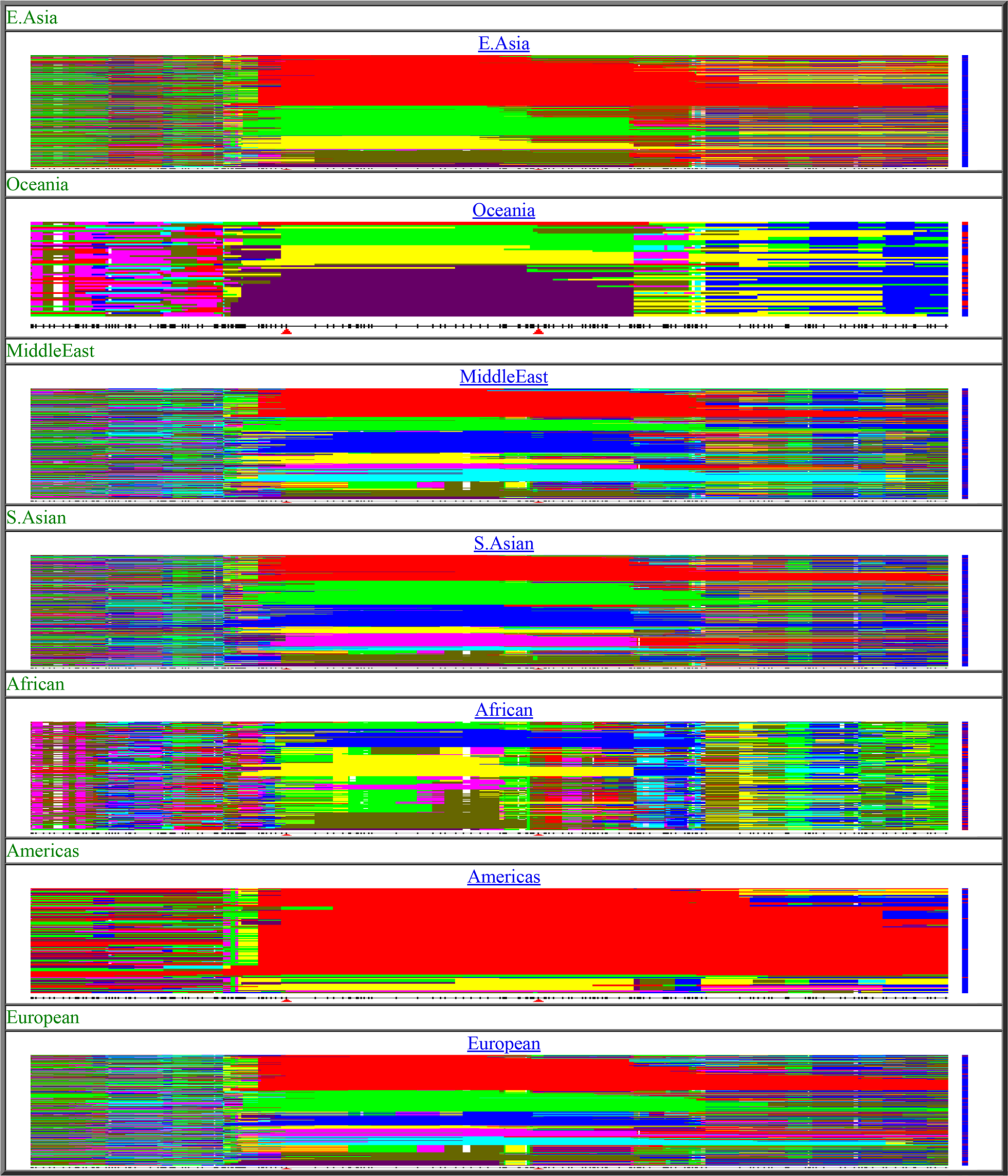
Continental and subcontinental haplotypes in a DNA region of 1 Mbps encompassing the protein C (*PROC*) gene. Rows and columns represent haplotypes and SNPs, respectively. Haplotypes of the same color are identical

To examine the diversity within continental populations, we used the Human Genome Diversity Project browser (http://hgdp.uchicago.edu/cgi-bin/gbrowse/HGDP/) and examined SNPs in a region of 1 Mbps encompassing the *PROC* gene—one of the 34 genes studied. As expected, the resulting haplotype chart (Fig. 5) shows a greater diversity in Africa compared with the situation in other continents or subcontinents. This greater diversity is characterized by complex patterns of African haplotypes shown as shorter multicolor haplotypes as compared to the longer haplotypes found in other world populations (Fig. 5), which is a result of lower degree of linkage disequilibrium in African haplotypes [17]. Haplotypes for individual populations included in this analysis are shown in Additional file 6: Fig. S3.

### Clinically important common hemostatic gene variants in ClinVar database

To examine the representation of populations with respect to the clinically relevant variants, we combined the drug response pharmacogenetic variants involved in thrombosis therapy and common pathogenic SNPs that alter function of hemostasis genes (n = 17), which we then investigated with Friedman’s two way analysis of variance by ranks. Friedman’s mean rank was largest in Europe and decreased with increasing genetic distance from Europe: EUR (4.4) > AMR (3.9) > SAS (3.8) > SOM (3.6) > YRI and EAS (2.7 each). This suggested that EUR was the best-represented population by the DNA variants investigated and that YRI and EAS were the least represented. There was a statistically significant difference in allele frequencies of the clinically relevant SNPs between the 6 populations, chi squared (Χ^2^) = 14.6, *P* = 0.012. Post hoc analysis with Wilcoxon signed-rank tests was conducted with a Bonferroni correction applied, which showed a significant difference between EUR and EAS (*P* < 0.05) and between YRI and EAS (*P* = 0.008).

**Fig. 6:**
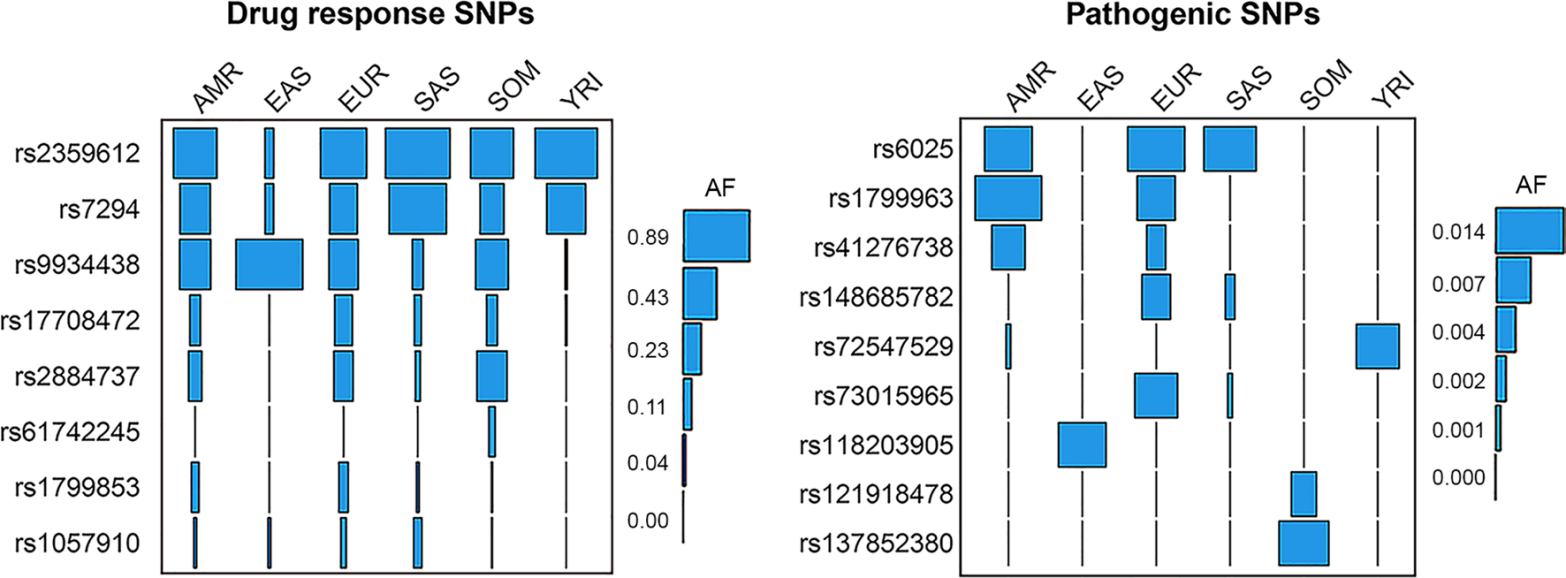
Battleship plots displaying allele frequencies (AF) for drug response (left) and pathogenic (right) SNPs in different populations. The horizontal width of the rectangle (or the square) is proportional to the magnitude of allele frequency. A legend for AF is shown on the right side of each plot with the highest AF on top and the lowest AF at the foot

Nine pathogenic hemostatic SNPs and eight drug response DNA variants with at least 1% AAF were identified in the populations studied (Fig. 6). In the battleship diagram shown in Fig. 6, the width of the rectangle, or the square, is proportional to the magnitude of the allele frequency. The wider the rectangle, the higher the allele frequency of a specific SNP in a population. The eight common hemostatic drug response SNPs shown in Fig. 6 (left) were identified in the *VKORC1* (rs2359612, rs7294, rs9934438, rs17708472, rs2884737, rs61742245) and in *CYP2C9* (rs1799853, rs1057910) genes. These eight variants are known to be associated with warfarin dose requirement [18]. One of these, rs61742245 (VKORC1 Asp36Tyr), was prevalent only in the SOM population. This variant is included in the 8% ‘C G C C G G A A’ haplotype in Table 1, as mentioned above, and is associated with warfarin resistance in the Horn of Africa [19–21] and, to a lesser extent, in the Middle East [22, 23]. The two genetically most distinct populations from Europe (YRI and EAS) had also the fewest number of drug response variants that reached the threshold of 1% AAF.

The EUR population appeared to have the most pathogenic SNPs reaching at 1% AAF (Fig. 6, right). In contrast, EAS and YRI populations had only one pathogenic variant each that reached the threshold 1% AAF. The variant *rs118203905* (FV R306T), also called Factor V Cambridge and associated with APC resistance [24], was common only in the EAS population. Another SNP, *rs41276738* (VWF R854Q) linked to type 2 N von Willebrand disease [25], reached at 1% AAF only in the AMR population. Further, SNPs *rs73015965* (PLG K38E) and rs148685782 (FGG A108G) associated with type I plasminogen deficiency [26] and reduced levels of fibrinogen [27], respectively, were common only in the EUR population. Two other common pathogenic SNPs were common only in the SOM population. One of these, *rs121918478* (AAF 0.0053; 1%), is a single nucleotide variant in factor II gene creating an amino acid substitution at position 461 of the protein (FII R461W), which causes hereditary factor II deficiency disease [28]. The other SNP found only in Somalia is the hemophilia A variant *rs137852380* (AAF 0.0105; 1%) in *F8* gene (FVIII G89D) [29]. Finally, a variant (rs72547529) previously associated with *VKORC1* loss of enzyme activity [30] was common only in the YRI population.

## Discussion

**Fig. 7:**
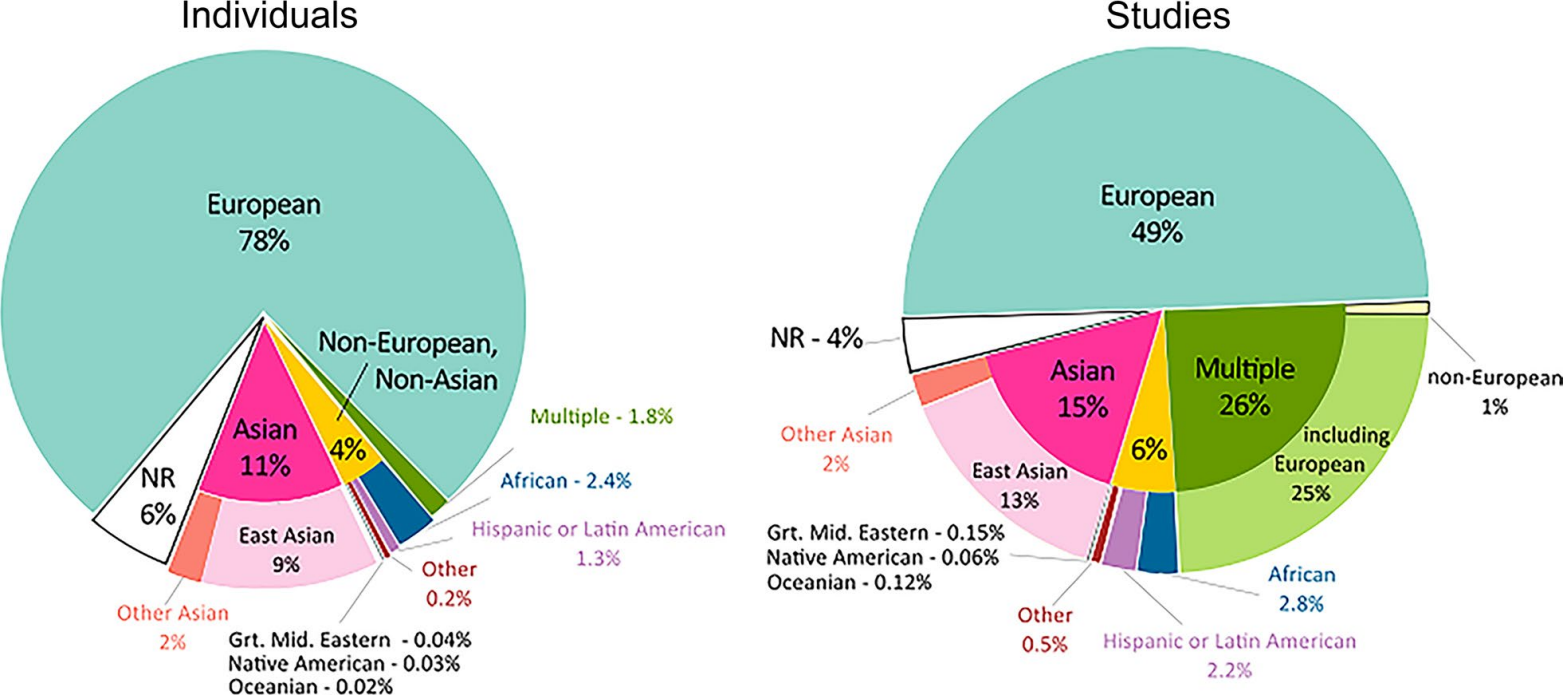
Ancestry category distribution in the GWAS Catalog. The figure shows the distribution of ancestry categories in percentages of individuals (N = 110,291,046; left panel) and studies (N = 4655; right panel). *Source*: https://www.ebi.ac.uk/gwas/docs/ancestry-data

Classic monogenic bleeding disorders of Mendelian inheritance style, such as hemophilia and von Willebrand disease, have been studied for decades. On the contrary, most genomics studies on drug response or on complex disorders such as thrombosis are more recent and have dramatically increased in the post-genomic era of the twenty-first century. In both cases, however, global ethnic populations are asymmetrically represented, with populations in wealthier Western countries representing an overwhelming majority of genetic studies found in the literature. For DNA variants of significance to thrombosis and hemostasis, present study confirms that notion and shows that the farther the genetic distance a population is from Europe the lesser is its representation in the ClinVar database when variants included in the Axiom PMRA were tested. For instance, while 11 of 17 pathogenic and drug response variants associated with hemostasis found in ClinVar were common in the EUR population, only five such variants were found in each of the EAS and YRI populations—the two genetically most distant populations from Caucasians in this study (Fig. 1). It is unlikely that the source of this discrepancy is because of European populations carrying more clinically significant hemostatic DNA variants than other populations. A more credible explanation is the fact that most of today's human genome research is based on DNA from populations who are predominantly European [31]. This view is supported by the data reported in the National Human Genome Research Institute (NHGRI) – European Bioinformatics Institute (EBI) Catalog of human genome-wide association studies (https://www.ebi.ac.uk/gwas). At the time of writing (August 10, 2021), 78% of all individuals and 49% of all studies in the NHGRI-EBI Catalog involve European populations (Fig. 7). By contrast, only 2.4% of the individuals and just 2.8% of the studies in NHGRI-EBI involve Africans although Africa hosts the greatest human genetic variation in the world [10, 32–35]. Such lopsided representation of world populations may give rise to misleading information in genetic databases such as ClinVar and OMIM, which assemble a large number of disease and pharmacogenetic markers based on studies carried on European populations. In addition, commercial SNP arrays such as the Axiom PMRA that were evaluated in this study employ DNA markers reported by such studies and, thus, are heavily Eurocentric despite claims to the contrary. Although pathogenic and drug response variants are expected to affect human populations in a similar manner, irrespective of ethnic attribute, lack of diversity in genomics research may lead to fewer such variants being detected in humans.

One limitation of this study is the limited number of common (≥ 1% AAF) pathogenic and drug response hemostatic variants identified on ClinVar database when SNPs included in the Axiom PMRA were employed. Yet, Friedman’s two way analysis of variance by ranks obviously showed statistically significant differences between the populations with respect to these clinically relevant variants. Interestingly, the pattern of Friedman’s mean ranks mirrored nearly perfectly the genetic shape shown in the PCA (Fig. 1) and changed with increasing genetic distance from Europeans, which suggested that the more genetically distant a population is from Europeans the lesser is its representation in the ClinVar database, at least as far as PMRA SNP array is concerned. This pattern could be also observed when allele frequencies of all 845 SNPs in each population were correlated with the mean allele frequencies for the 6 populations. The best correlations with the mean allele frequencies were provided by Europeans and those populations with genetic proximity to Europeans, whereas Yoruba and East Asians presented the lowest correlations with the mean allele frequencies.

Another limitation is the merging of non-African continental and subcontinental populations. Obviously, it might be expected that there is some variation in allele frequencies within a particular continental or subcontinental population. The variation in gene frequencies, however, is much greater within African populations as we observed in this study between the Somali and other African populations when analyzed the *VKORC1* haplotypes. Indeed, *VKORC1* haplotypes in Somalis were closer to the Toscani population (TSI) in Italy rather than to Luhya in Webuye, Kenya, despite the latter country being a neighbor to Somalia. Yet, Horn of African populations and other ethnolinguistic groups in Africa are not represented in large genome ventures such the 1000 genomes.

Finally, we would like to discuss the use of haplotype information in the field of medical genetics. First, the importance and usefulness of haplotyping is well established through traditional linkage analysis in families with inherited disease. Second, there are also other circumstances where genetic phasing plays a role. For example, hematopoietic stem cell transplants using haplotype matched recipients perform better clinically than those using allele matched but haplotype mis-matched patients [36]. To mention another example that applies methods akin to our own work, an interesting new proposal appeared recently in the field of pre-implantation genetic diagnosis (PGD) for molecular disorders. This requires construction of parental haplotypes. Classically, haplotype resolution is obtained by genotyping several polymorphic markers in both parents and at least one additional relative. Zeevi et al. [37] performed targeted sequencing of *CFTR* gene variants and flanking polymorphic SNPs on individuals from PGD families. Heterozygous calls in each individual were phased using massive parallel sequencing genomic data for reference populations. For founder mutation carriers, the haplotyping approach facilitated near perfect phasing accuracy. Their results demonstrate the feasibility of replacing classical haplotype phasing with population-based phasing, thus eliminating the need for recruiting additional family members in the PGD procedure.

In precision medicine, by definition, it is expected that many diagnostic tests for selection of appropriate therapies will be based on the patient's genetic characteristics. Skewed representation of global populations in genetic databases affects the effectiveness of such endeavors, especially when modelled on interfering anti-sense oligonucleotides. For example, one could speculate of miRNAs as potential cancer biomarkers and therapeutic targets. However, the breakthrough of circulating miRNA-based cancer therapy has yet to come.

In this study, our results demonstrate the importance of considering ethnicity when prescribing warfarin for the prophylaxis and treatment of venous thrombosis and pulmonary embolism. Warfarin has a very narrow therapeutic range. If the dose is too high, the patient may be at risk of serious bleeding and if the dose is too low there is an increased risk of thrombosis. Pharmacogenetic testing of the *VKORC1* rs61742245 polymorphism to control warfarin resistance may be considered as a way to improve the safety and efficacy of warfarin use but, unfortunately, there is limited evidence to support routine testing due to the even greater impact of diet, possible lack of compliance, and environmental factors on the warfarin levels monitored in blood. However, as a proof of principle, our *VKORC1* example shows the power of a population-based phasing approach when applied to GWAS data. The basis for such analysis is linkage disequilibrium of polymorphisms in the vicinity of disease-causing founder mutations, including pharmaco-genetically important variants.

## Conclusions

In conclusion, this study highlights the importance of diversity in genomics research pertaining to DNA variants of clinical importance to thrombosis and hemostasis. By including more African, Asian and indigenous ethnic groups in genomics studies, more clinically relevant hemostatic variants of significance to thrombosis and bleeding disorders would presumably be discovered and reported to the different clinical genetic databases as well as be included in different commercial SNP arrays. Haplotype-based methods offer a powerful approach to disease gene mapping, based on the association between causal mutations and the ancestral haplotypes on which they arose [17]. We confirm here that the haplotypes of a single gene (*VKORC1*) based on only 8 SNPs are highly correlated across populations and could be used not only for population studies but also for disease gene mapping.

## Authors' information

**Abdimajid Osman** is Associate Professor of Medical Genetics and senior Clinical Scientist at Linköping University Hospital. **Jon Jonasson** is Associate Professor of Clinical Genetics and senior consultant Clinical Geneticist at Linköping University Hospital.

## Supplementary Information

**Additional file 1: Table S1.** A list of 845 SNPs in genes associated with hemostasis. ClinVar annotations are shown on the column farthest to the right.

**Additional file 2: Fig. S1.** Principal component analysis of 849,267 high-quality markers that were included in the Axiom Precision Medicine Research Array. The Somali data were projected onto PC1 and PC2 data from PGG population (https://www.pggpopulation.org/) as we previously described [11].

**Additional file 3: Table S3.** Pearson correlations for 845 haemostatic gene variants with at least 1% MAF in at least 1 population.

**Additional file 4: Fig. S2.** Population multiplot for 845 hemostatic gene variants. Allele frequencies are shown on the axis.

**Additional file 5: Table S2.** Multi-SNP haplotype phasing for hemostatic genes constructed with the Shapeit v2 method. Each tab in the Excel file shows haplotypes for one hemostatic gene.

**Additional file 6: Fig. S3.** Population haplotypes in a region of 1 cM (1 Mbp) encompassing the *PROC* gene. Rows and columns represent haplotypes and SNPs, respectively. Haplotypes of the same color are identical.

## Data Availability

Data for the Somali population are available at the Array Express database (https://www.ebi.ac.uk/arrayexpress) under the accession number: E-MTAB-8331, as we previously described [11]. Data for other human populations were collected from the 1000 genome project [8].

## Abbreviations

AAF: Alternative allele frequencies
SOM: Somali in Northeastern Somalia
LWK: Luhya in Webuye, Kenya
YRI: Yoruba in Ibadan, Nigeria
ASW: African Americans in Southwest USA
AMR: Ad-mixed American
CHB: Han Chinese in Beijing, China
JPT: Japanese in Tokyo, Japan
SAS: East Asian
CEU: Northern Europeans from Utah
TSI: Toscani in Italy
EUR: European
GIH: Gujarati Indians in Houston, Texas, USA
SAS: South Asian
PGD: Pre-implantation genetic diagnosis
