# Supplementary material for: Cross-ethnic analysis of common gene variants in hemostasis show lopsided representation of global populations in genetic databases
Source: BMC Med Genomics. 2022 Mar 25;15:69. doi: 10.1186/s12920-022-01220-0 (PMC8957123; doi:10.1186/s12920-022-01220-0)
Supplement: Supplementary file 2 — Additional file 2: Fig. S1. Principal component analysis of 849,267 high-quality markers that were included in the Axiom Precision Medicine Research Array. The Somali data were projected onto PC1 and PC2 data from PGG population (https://www.pggpopulation.org/) as we previously described [11]. [file 12920_2022_1220_MOESM2_ESM.pdf]

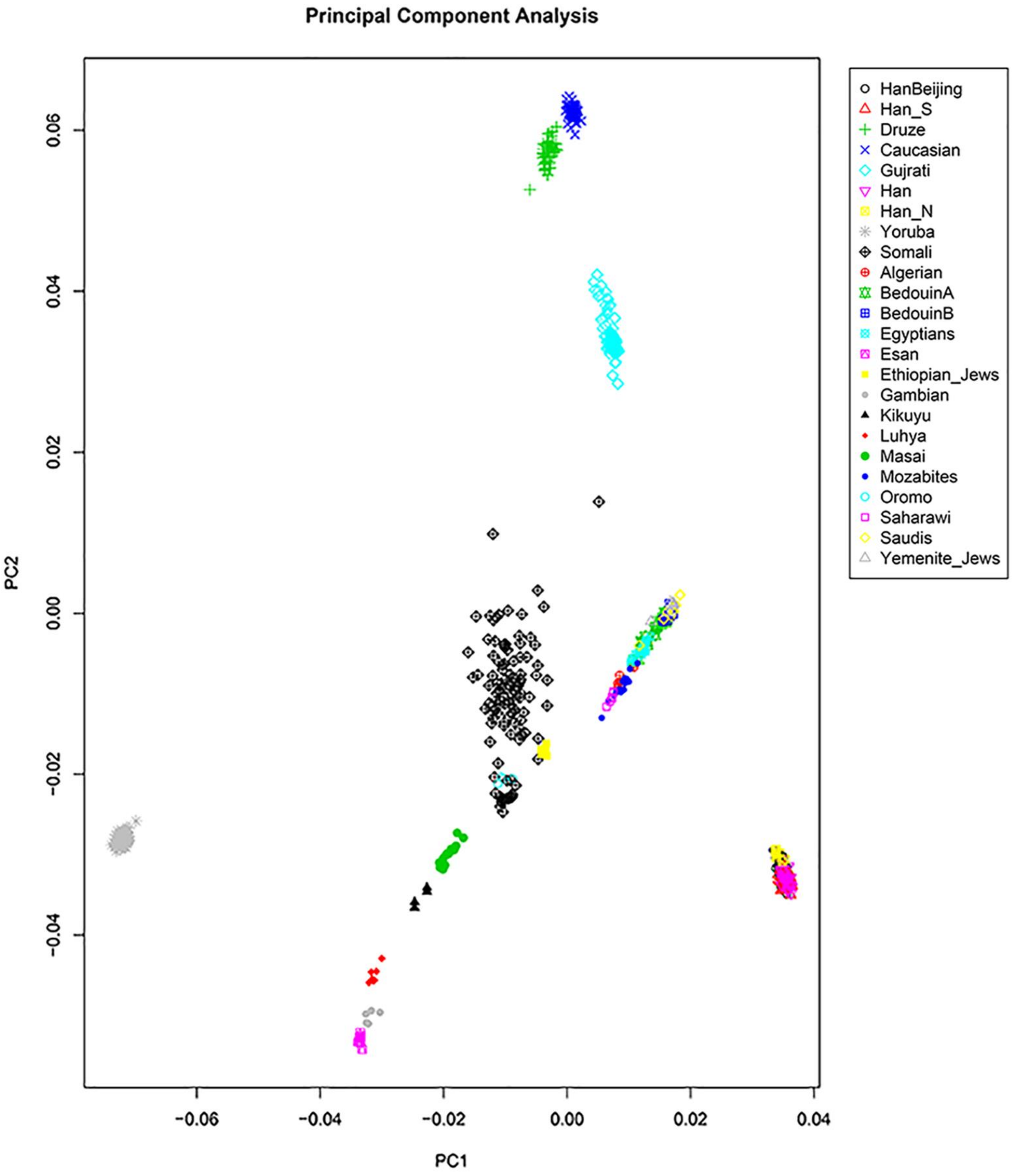

Supplementary Figure S1. Principal component analysis of 849,267 high-quality markers that were included in the Axiom Precision Medicine Research Array. The Somali data were projected onto PC1 and PC2 data from PGG population (https://www.pggpopulation.org/) as we previously described (Ali, A.A., Aalto, M., Jonasson, J., Osman, A. Genome-wide analyses disclose the distinctive HLA architecture and the pharmacogenetic landscape of the Somali population. Sci Rep 10, 5652 (2020). https://doi.org/10.1038/s41598-020-62645-0).
