# Supplementary material for: Cross-ethnic analysis of common gene variants in hemostasis show lopsided representation of global populations in genetic databases
Source: BMC Med Genomics. 2022 Mar 25;15:69. doi: 10.1186/s12920-022-01220-0 (PMC8957123; doi:10.1186/s12920-022-01220-0)
Supplement: Supplementary file 3 — Additional file 3: Table S3. Pearson correlations for 845 haemostatic gene variants with at least 1% MAF in at least 1 population. [file 12920_2022_1220_MOESM3_ESM.pdf]

Supplementary Table S3. Pearson correlations for 845 haemostatic gene variants with at least 1% MAF in at least 1 population.

	AMR	EAS	EUR	SAS	SOM	YRI
**AMR**		0.904	0.964	0.957	0.905	0.817
**EAS**	0.904		0.862	0.899	0.805	0.722
**EUR**	0.964	0.862		0.955	0.909	0.806
**SAS**	0.957	0.899	0.955		0.901	0.812
**SOM**	0.905	0.805	0.909	0.901		0.910
**YRI**	0.817	0.722	0.806	0.812	0.910	

Max **Min**
