# Supplementary material for: Cross-ethnic analysis of common gene variants in hemostasis show lopsided representation of global populations in genetic databases
Source: BMC Med Genomics. 2022 Mar 25;15:69. doi: 10.1186/s12920-022-01220-0 (PMC8957123; doi:10.1186/s12920-022-01220-0)
Supplement: Supplementary file 4 — Additional file 4: Fig. S2. Population multiplot for 845 hemostatic gene variants. Allele frequencies are shown on the axis. [file 12920_2022_1220_MOESM4_ESM.pdf]

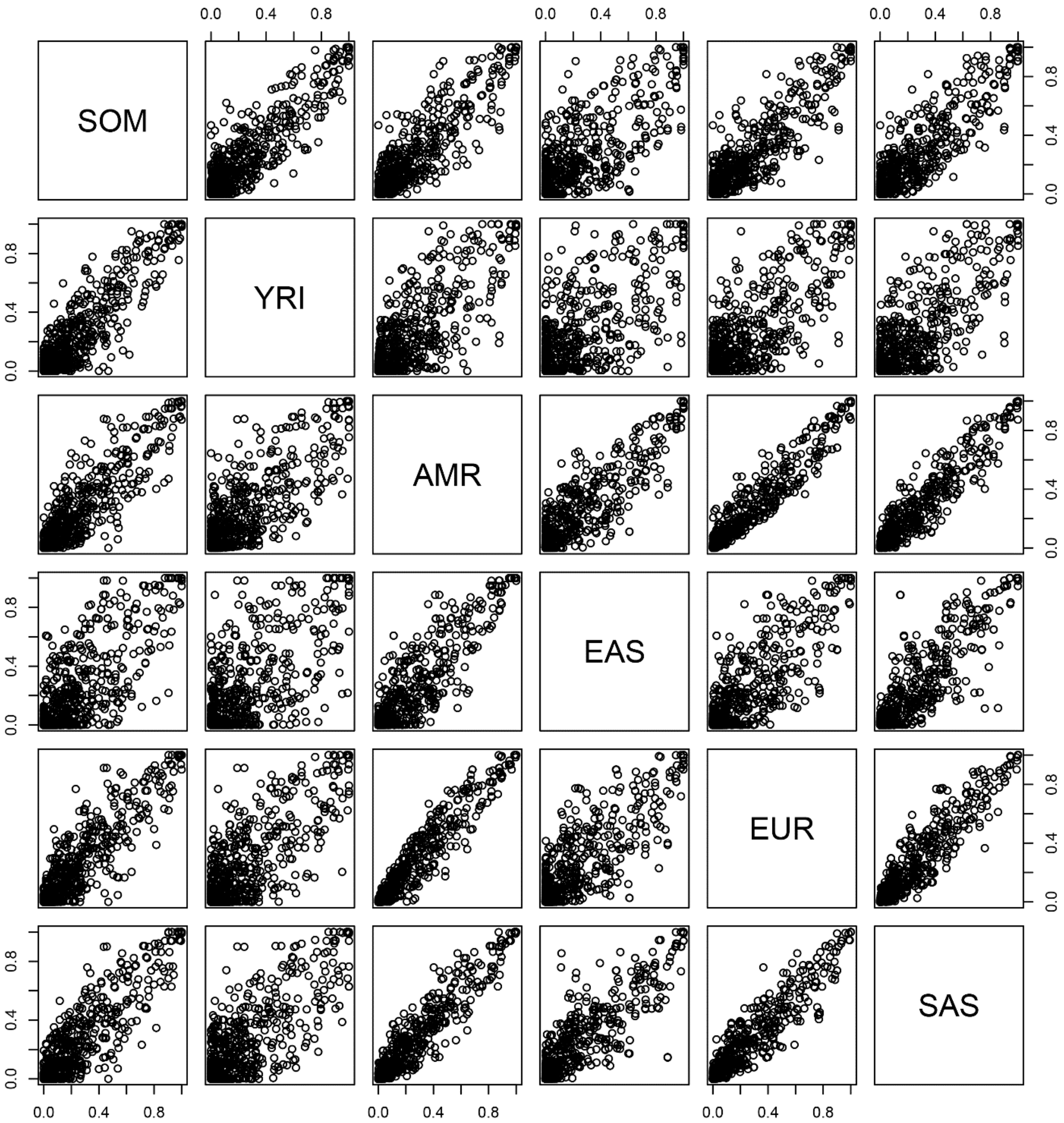

Supplementary Figure S2. Population multiplot for 845 hemostatic gene variants. Allele frequencies are shown on the axis.
