# Supplementary material for: Cross-ethnic analysis of common gene variants in hemostasis show lopsided representation of global populations in genetic databases
Source: BMC Med Genomics. 2022 Mar 25;15:69. doi: 10.1186/s12920-022-01220-0 (PMC8957123; doi:10.1186/s12920-022-01220-0)
Supplement: Supplementary file 6 — Additional file 6: Fig. S3. Population haplotypes in a region of 1 cM (1 Mbp) encompassing the PROC gene. Rows and columns represent haplotypes and SNPs, respectively. Haplotypes of the same color are identical. [file 12920_2022_1220_MOESM6_ESM.pdf]

European

Russian

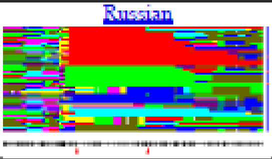

French

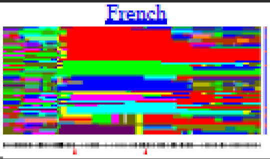

Tuscan

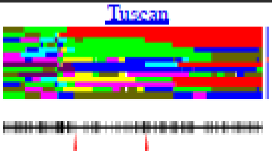

Sardinian

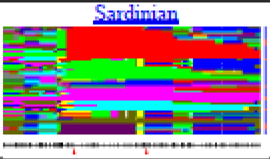

Basque

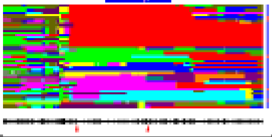

Adygei

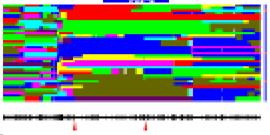

Orcadian

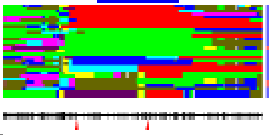

Italian

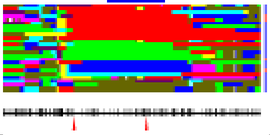

MiddleEast

Bedouin

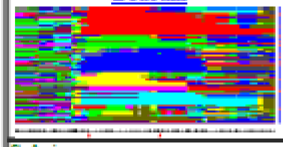

Mozabite

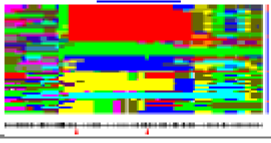

Palestinian

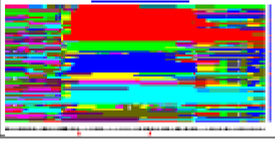

Druze

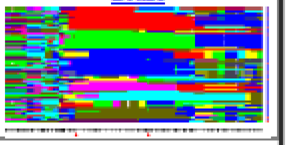

S.Asian

Balochi

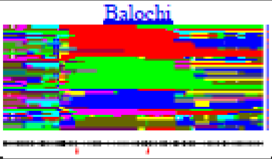

Burusho

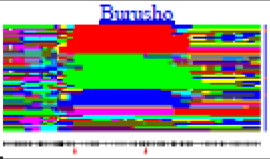

Uygur

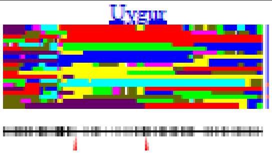

Sindhi

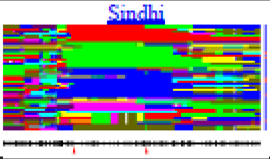

Hazara

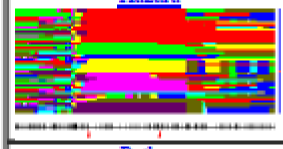

Brahui

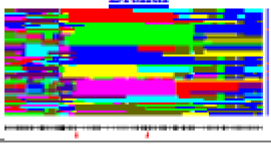

Makrani

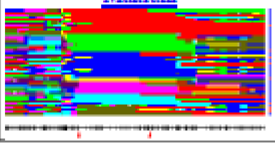

Kalash

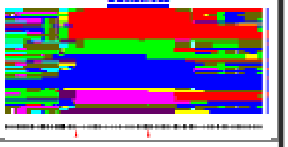

Pathan

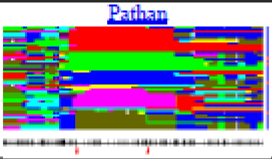

E.Asia

Mongola

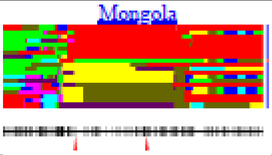

Hezhen

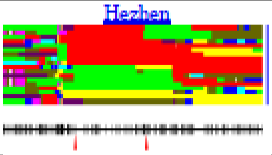

Lahu

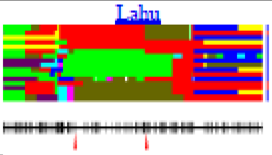

Cambodian

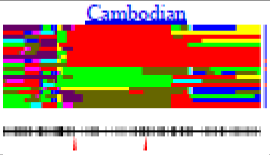

Oroqen

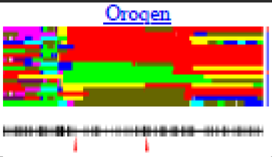

Dai

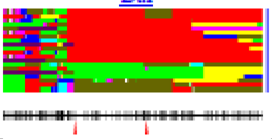

Tujia

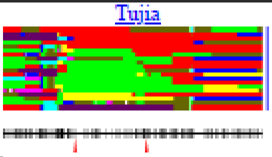

Han

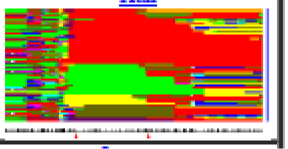

[illegible]

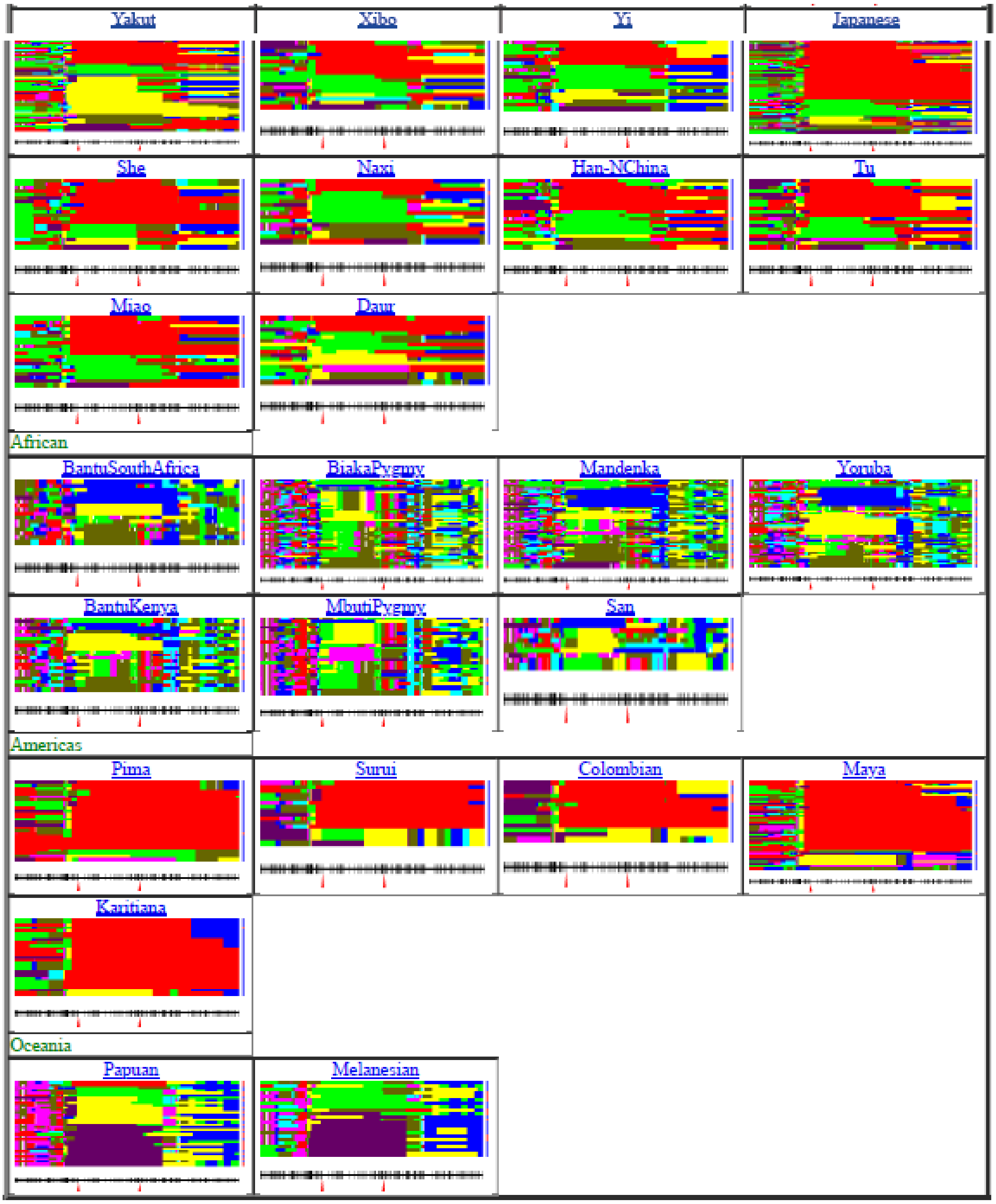

Supplementary Figure S3. Population haplotypes in a region of 1 cM (1 Mbp) encompassing the *PROC* gene. Rows and columns represent haplotypes and SNPs, respectively. Haplotypes of the same color are identical
